# Myocarditis after COVID‐19 mRNA vaccination in Australia

**DOI:** 10.5694/mja2.51657

**Published:** 2022-07-13

**Authors:** Suraj K Varma, Ari E Horton, Anna L Taylor, Michael R Ditchfield, Sarah A Hope, Srinidhi JV Rao

**Affiliations:** ^1^ MonashHeart Melbourne VIC; ^2^ Monash Children’s Hospital Melbourne VIC; ^3^ Monash Cardiovascular Research Centre Monash University Melbourne VIC; ^4^ Victorian Heart Institute Monash University Melbourne VIC; ^5^ Monash Imaging, Monash Health Melbourne VIC; ^6^ Monash University Melbourne VIC

**Keywords:** COVID‐19, Magnetic resonance imaging, Echocardiography, Vaccination, Cardiac imaging techniques

Myocarditis in adolescents and young adults following the administration of coronavirus disease 2019 (COVID‐19) mRNA vaccines has been reported.^1^ Vaccination of 12–16‐year‐old adolescents with Comirnaty (tozinameran, Pfizer–BioNTech) and Spikevax (elasomeran, Moderna) was approved in Australia on 22 July 2021 and 3 September 2021 respectively.^2^ In this report, we describe the initial diagnosis, imaging findings, and short term outcomes for adolescents who presented with COVID‐19 vaccine‐associated myocarditis to the Monash Children’s Hospital, a tertiary centre in Melbourne with a paediatric cardiology service. The Monash Health human research ethics committee (QA/81618/MonH‐2021‐291293) approved the study.

We included adolescents (12–18 years old) who presented with typical symptoms of myocarditis associated with troponin rise (> 15 ng/L) within 28 days of first or second doses of COVID‐19 mRNA vaccines during 1 August – 31 December 2021. Myocarditis was defined by standard criteria.^3^ All patients underwent electrocardiography (ECG), echocardiography, and cardiac magnetic resonance (CMR) imaging. CMR images (T2 and late gadolinium enhancement imaging sequences) were acquired with a 1.5 T scanner (Avanto‐Fit, Siemens Healthineers; 32 patients) or a 3 T scanner (Ingenia, Philips Healthcare; one patient). All patients were followed up (as outpatients) two to three weeks after their initial presentation.

None of the 33 included patients presented with congestive heart failure or required intensive care treatment, inotropic support, or immunoglobulin or steroid therapy (Box 1). Fourteen patients (42%) had rising troponin levels at presentation; eight had ECG changes typical for pericarditis, but no arrhythmias were detected by inpatient telemetry. Left ventricular systolic function was normal at presentation in 29 patients and mildly impaired in four (Box 2), and was normal in all patients by follow‐up. Tissue Doppler velocity, a marker of cardiac function, was normal for the thirty patients with technically satisfactory data; the median global longitudinal strain value was normal (20%; interquartile range [IQR], 18.5–20%).

Box 1 **Presentation characteristics of 33 adolescents with COVID‐19 vaccine‐associated myocarditis, Melbourne, 1 August – 31 December 2021**

**Table:** 

Characteristic	Number/value
Patients	33
Sex	
Boys	27 (82%)
Girls	6 (18%)
Age (years), median (IQR)	14.6 (13.0–16.4)
COVID‐19 vaccine	
Pfizer–BioNTech	28 (85%)
Moderna	5 (15%)
Vaccine dose	
First	6 (18%)
Second	27 (82%)
Vaccination to presentation (days)	
Median (IQR)	3 (3–4.5)
Range	2–26
Symptoms	
Chest pain	33 (100%)
Fever	4 (12%)
Shortness of breath	2 (6%)
Headache	7 (21%)
Myalgia	6 (18%)
Palpitations	3 (9%)
Other symptom	6 (18%)
Length of hospital stay (days), median (IQR)	2.3 (1.9–3.0)

COVID‐19 = coronavirus disease 2019; IQR = interquartile range.

Box 2 **Clinical characteristics of 33 adolescents with COVID‐19 vaccine‐associated myocarditis, Melbourne, 1 August – 31 December 2021**

**Table:** 

Characteristic	Number/value
**Biochemistry**	
Troponin, peak level (ng/L), median (IQR)	2837 (1181–7836)
C‐reactive protein* (mg/L), median (IQR)	16 (6.5–44.5)
**Electrocardiography**	
Normal	25 (76%)
Significant ST elevation	6 (18%)
T wave changes	2 (6%)
Left ventricular ejection fraction	
Normal (≥ 55%)	29 (88%)
Mild dysfunction (45–54%)	4 (12%)
**Cardiac magnetic resonance imaging (CMR) (diagnostic)**	
Admission to CMR (days), median (IQR)	2 (1–2.5)
Myocardial oedema	24 (73%)
Late gadolinium enhancement^†^	27 (84%)
Pericardial effusion (small/trace)	25 (76%)
Pericardial enhancement	2 (6%)
Lake Louise criteria for myocarditis^4^	22 (69%)
CDC case definition of myocarditis^1^ ^,^ ^†^	
Confirmed	22 (69%)
Probable	10 (31%)

CDC = Centers for Disease Control and Prevention (United States); IQR = interquartile range.

* Reference interval: < 5 mg/L; data available for thirty patients only, for 25 of whom the value exceeded the reference interval.

† Data available for thirty‐two patients only.

CMR imaging was performed early in most admissions (median, 2 days; IQR, 1–2.5 days); the median interval between peak troponin level and CMR was one day (IQR, 1–2 days). CMR findings were abnormal in 27 of 32 patients (contrast medium could not be administered to one patient because of anxiety), showing late gadolinium enhancement in a patchy subepicardial to transmural pattern, and was especially marked in the inferolateral left ventricular free wall (Box 3). Evidence of oedema in corresponding segments was detected by T2‐weighted CMR in 22 of 32 patients, meeting the Lake Louise criteria for myocarditis.^4^ The right ventricular apex was affected in isolation in one patient. A small or trace pericardial effusion was noted in 25 of 33 patients, and pericardial enhancement in two of 33 patients. Myocarditis (CDC criteria^1^) was confirmed for 22 of 32 patients and was probable for ten of 32 patients.

Box 3 **Late gadolinium enhancement in mid‐anterolateral and mid‐inferolateral myocardial segments**

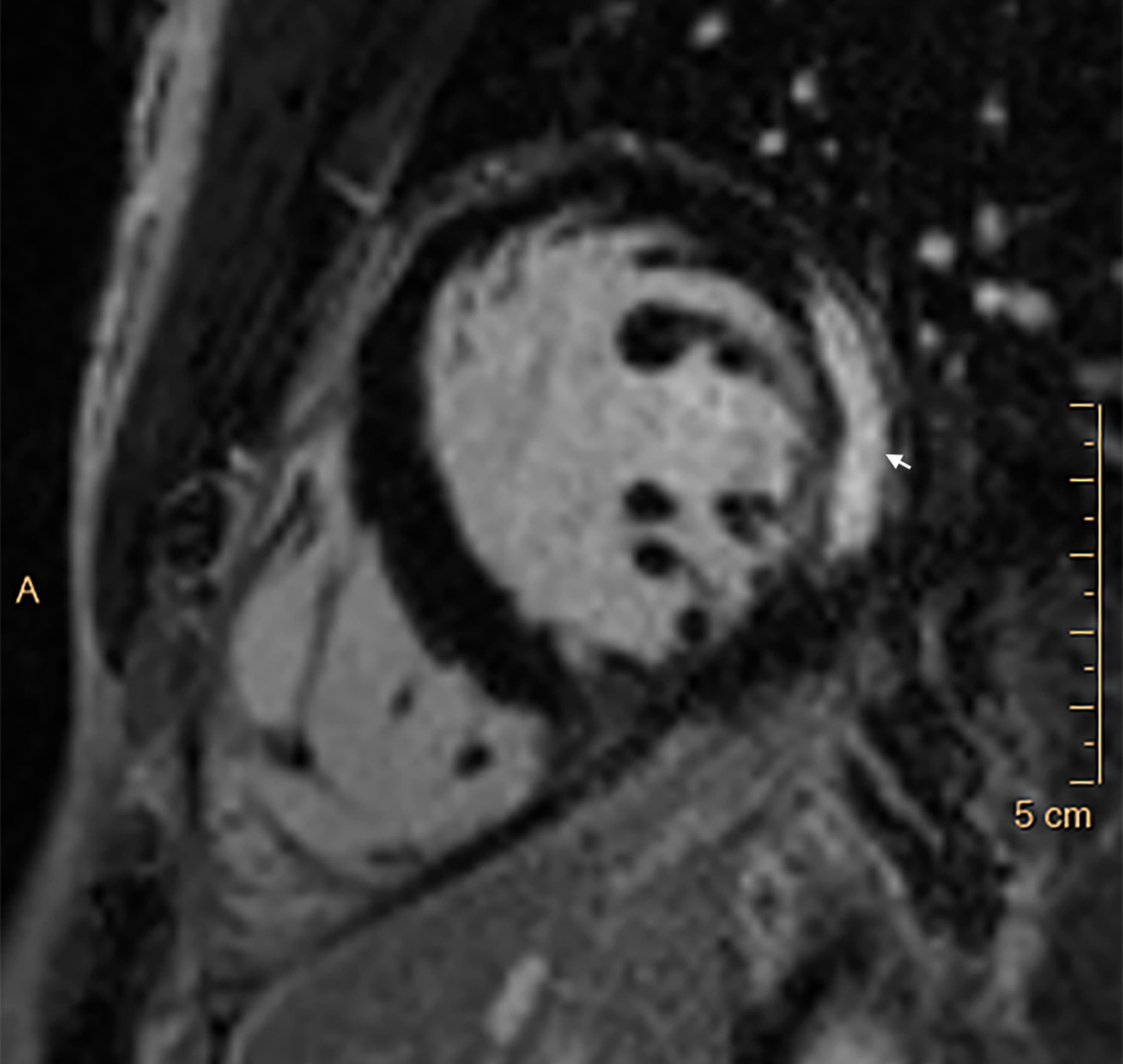

A = anterior; arrow: area of late gadolinium enhancement. ◆

Thirty‐two patients improved in hospital after treatment with high dose ibuprofen (10 mg/kg/dose; typical, 6–8 × 600 mg/dose [maximum, 2400 mg/day]) for one week or until symptom resolution and proton pump inhibitor therapy (omeprazole, 20 mg daily). The median hospital stay was 2.3 days (IQR, 1.9–3.0 days). Two weeks’ bed rest was recommended to all patients, followed by a gradual return to normal activities over three months.

Our vaccine‐associated myocarditis study is the largest reported for a single children’s hospital. Only one of our participants had a history of prior SARS‐CoV‐2 infection, so background immunity is unlikely to have influenced the adverse event profile of vaccination. COVID‐19 mRNA vaccine‐associated myocarditis has a mild, self‐resolving clinical course, in contrast to reported complications and long term sequelae associated with COVID‐19, such as multisystem inflammatory syndrome in children, and other forms of myocarditis.^5^, ^6^ The long term consequences of myocardial injury with vaccine‐associated myocarditis nevertheless warrant further investigation.

## Open access

Open access publishing facilitated by Monash University, as part of the Wiley – Monash University agreement via the Council of Australian University Librarians.

## Competing interests

No relevant disclosures.
